# More than simulation: the HelpMeSee approach to cataract surgical training

**Published:** 2023-12-01

**Authors:** Van Charles Lansingh, Akshay Gopinathan Nair

**Affiliations:** 1 Chief Medical Officer: HelpMeSee, New York, USA.; 2 Clinican-Scientist/Consultant: HMS Vision Pvt Ltd, Powai Hiranandani, Mumbai, India.

**Despite the many benefits of virtual reality surgical simulation, trainees still benefit from pre-learning and the presence of experienced instructors.**

**Figure:**
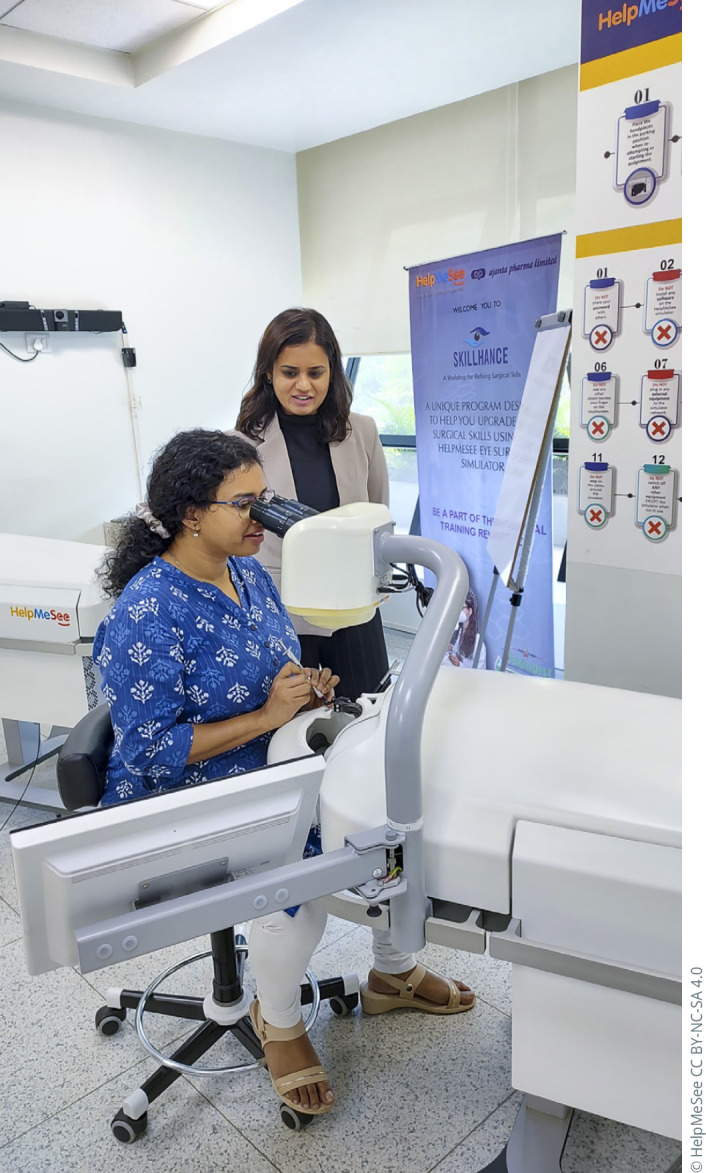
An instructor training a resident on the HelpMeSee surgical simulator. **INDIA**

Simulation in ophthalmology is not a new concept. The use of animal eyes, vegetative material, and inanimate objects to learn and practice surgical skills has been around for many years, allowing trainees to practice surgical techniques in a safe and controlled environment.^1^

Virtual reality (VR) ophthalmic surgery simulators are able to provide a surgical training environment that is visually realistic (Figure 1) and includes built-in haptic (motorsensory) feedback systems; these create a realistic experience by mimicking the complexities of real-life surgery.

**Figure 1:**
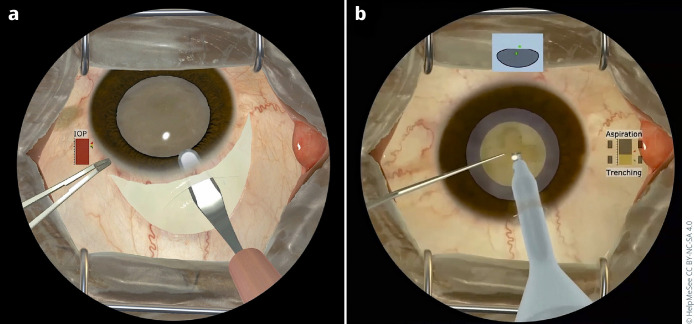
**(a)** The view through the eyepieces of the simulator – seen while creating a sclerocorneal tunnel in manual small incision cataract surgery (MSICS). **(b)** The view while performing trenching in phacoemulsification.

HelpMeSee, a non-profit organisation with headquarters in the USA, has developed an eye surgery simulator that is specifically designed to support the cataract surgery training courses offered at its training centres in India, France, the USA, China, and Madagscar. The courses include manual small-incision surgery, phacoemulsification, suturing, and complications management. Each course includes self study, classroom sessions, hands-on laboratory exercises (depending on the course), and instructor-led simulation-based training and feedback.

To make training available and affordable, HelpMeSee offers subsidised training in MSICS in India, China, and Madagascar.

## Key principles in HelpMeSee simulation-based training

There is an old adage in training. “A simulator simulates, it does not train.” A comprehensive pre-reading resource, experienced instructors, and individualised, objective assessment of surgeons’ performance are important components of the HelpMeSee simulation-based training curriculum. Our experience has shown that each of these components play crucial roles in a trainee's learning journey.

### 1. Pre-reading and assessment

On enrolment, before the start of the course, every trainee is given access to an interactive e-book that is available both online and offline. The e-book begins with an overview of the anatomy of the eye, the surgical instruments needed, and the surgical steps. Next, it demonstrates each step using high quality videos and animations that are accompanied by audio commentary from expert surgeons, who also give tips on how to avoid complications. There is a test at the end of each chapter that trainees must pass before the programme starts; this ensures that everyone on the course has a solid theoretical understanding before the practical training begins.

“The constant presence of an instructor by the side of someone undergoing training on the simulator is invaluable.”

### 2. Instructor-led training

External feedback has always been thought to be crucial to technical skill development in novice surgeons,^2^ and it has been demonstrated that verbal feedback from an expert instructor leads to lasting improvements in performance. Therefore, all simulator-based training courses offered at HelpMeSee are led by an instructor who has been specially trained to provide this type of training.

The constant presence of an instructor by the side of someone undergoing training on the simulator is invaluable. The instructor can demonstrate the steps in the beginning, observe the trainee, give immediate feedback on crucial aspects such as instrument holding and surgical technique, and provide insightful comments on how to avoid complications.

The instructor is also responsible for assessing trainees' surgical competency on the simulator at the end of training.

### 3. Supervised practice and individualised feedback

The course includes sessions of supervised practice on the simulator, followed by feedback or debriefing sessions at the end of each day of training. Here, the instructor reviews the performance of each trainee, giving individuals positive feedback about tasks that were done well, feedback about what went wrong, and advice on how to improve.

### 4. Simulator-supported learning

**Figure 2:**
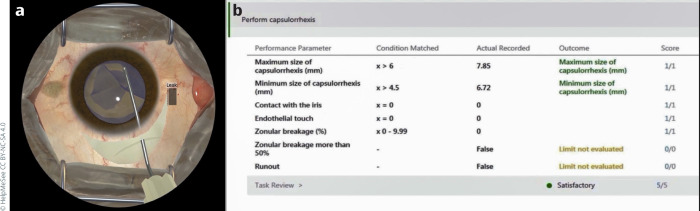
**(a)** A capsulorrhexis being created through the sclerocorneal tunnel in MSICS. **(b)** After completion of the capsulorrhexis, the system displays the performance parameters that were used to assess the attempt. An objective scoring tool evaluates each attempt and grades it as either satisfactory or unsatisfactory, based on the statistics listed under Actual Recorded’ and the number of errors made.

The HelpMeSee eye surgical simulator software assesses surgical performance by comparing it with objective parameters (Figure 2) and competency assessment rubrics. This provides an objective way for instructors to understand trainees’ performance and to qualify and assess their competency.

The system also supports trainees by offering immediate feedback during the prodecure (e.g., by providing a real-time alert when a trainee makes an error). Trainees can view a video recording of each task they perform on the simulator, which is an opportunity for **reflective learning**: trainees can watch the video and reflect on their performance and on how to improve.

## The benefits

Several studies have been published on the validity and efficacy of virtual-reality-based cataract surgical simulators. One found that trainees who used a simulator to practice surgical techniques demonstrated significant improvements in both their surgical skills and confidence levels compared to those who did not use the simulator.^3^ Other studies have consistently demonstrated that a surgical simulator is an effective training tool that can improve surgical performance and confidence levels.^4^ In a randomised controlled trial, it was demonstrated that simulation based training on the HelpMeSee eye surgery simulator prior to live cataract surgery reduced the number of errors in the first twenty live operations that trainees performed.^5,6^

To find out more about HelpMeSee's training courses, reach out to training@helpmesee.org.
